# Dual Opposing Roles of Metallothionein Overexpression in C57BL/6J Mouse Pancreatic β-Cells

**DOI:** 10.1371/journal.pone.0137583

**Published:** 2015-09-03

**Authors:** Suqin Chen, Junying Han, Yeqi Liu

**Affiliations:** 1 The Research Institute for Children, Children’s Hospital at New Orleans, New Orleans, Louisiana, United States of America; 2 Department of Medical Genetics, Zhongshan Medical College, Sun Yat-sen University, Guangzhou, Guangdong Province, People’s Republic of China; 3 Department of Pediatrics, Louisiana State University Health Sciences Center, New Orleans, Louisiana, United States of America; Communaute d\'Universites et d\'Etablissements Lille Nord de France, FRANCE

## Abstract

**Background:**

Growing evidence indicates that oxidative stress (OS), a persistent state of excess amounts of reactive oxygen species (ROS) along with reactive nitrogen species (RNS), plays an important role in insulin resistance, diabetic complications, and dysfunction of pancreatic β-cells. Pancreatic β-cells contain exceptionally low levels of antioxidant enzymes, rendering them susceptible to ROS-induced damage. Induction of antioxidants has been proposed to be a way for protecting β-cells against oxidative stress. Compared to other antioxidants that act against particular β-cell damages, metallothionein (MT) is the most effective in protecting β-cells from several oxidative stressors including nitric oxide, peroxynitrite, hydrogen peroxide, superoxide and streptozotocin (STZ). We hypothesized that MT overexpression in pancreatic β-cells would preserve β-cell function in C57BL/6J mice, an animal model susceptible to high fat diet-induced obesity and type 2 diabetes.

**Research Design and Methods:**

The pancreatic β-cell specific MT overexpression was transferred to C57BL/6J background by backcrossing. We studied transgenic MT (MT-tg) mice and wild-type (WT) littermates at 8 weeks and 18 weeks of age. Several tests were performed to evaluate the function of islets, including STZ *in vivo* treatment, intraperitoneal glucose tolerance tests (IPGTT) and plasma insulin levels during IPGTT, pancreatic and islet insulin content measurement, insulin secretion, and islet morphology assessment. Gene expression in islets was performed by quantitative real-time PCR and PCR array analysis. Protein levels in pancreatic sections were evaluated by using immunohistochemistry.

**Results:**

The transgenic MT protein was highly expressed in pancreatic islets. MT-tg overexpression significantly protected mice from acute STZ-induced ROS at 8 weeks of age; unexpectedly, however, MT-tg impaired glucose stimulated insulin secretion (GSIS) and promoted the development of diabetes. Pancreatic β-cell function was significantly impaired, and islet morphology was also abnormal in MT-tg mice, and more severe damage was detected in males. The unique gene expression pattern and abnormal protein levels were observed in MT-tg islets.

**Conclusions:**

MT overexpression protected β-cells from acute STZ-induced ROS damages at young age, whereas it impaired GSIS and promoted the development of diabetes in adult C57BL/6J mice, and more severe damage was found in males.

## Introduction

Hyperglycemia is a hallmark of diabetes, resulting from absolute or relative insulin deficiency. As insulin-producing cells, the dysfunction of pancreatic β-cells is considered as a major factor contributing to the development of both type 1 diabetes (T1D) and type 2 diabetes (T2D). T1D is characterized by the absence of insulin in pancreatic β-cells caused by autoimmune reaction. T2D, on the other hand, is mainly caused by the failure of pancreatic β-cells to secrete sufficient insulin to overcome insulin resistance, finally resulting in diabetes. Persistent hyperglycemia leads to secondary complications in the peripheral tissues including eyes, nerves, kidneys, and vascular tissues. Persistent hyperglycemia also impairs pancreatic β-cells, leading to glucose desensitization, β-cell exhaustion, and glucose toxicity [1]. Oxidative stress (OS), a persistent state of excessive production of reactive oxygen species (ROS) along with reactive nitrogen species (RNS), has been proposed to be a contributor to the failure of pancreatic β-cells, insulin resistance, and diabetic complications [1, 2]. As a byproduct of normal fuel metabolism, a low level of ROS has been proposed to be a signal for mediating glucose-stimulated insulin secretion (GSIS) and insulin signaling in pancreatic β-cell differentiation and survival [3–5]. However, excessive ROS/RNS leads to cellular macromolecular damage in proteins, lipids, carbohydrates, and nucleic acids. To protect against ROS/RNS, cells have evolved endogenous defense systems that can be divided into two major categories: enzymes [e.g., superoxide dismutases (SOD), catalase, and glutathione peroxidase (GPx)] and non-enzymatic systems (e.g., glutathione GSH, and vitamins C, and E). Unfortunately, pancreatic islets contain exceptionally low levels of antioxidant enzymes, rendering them more sensitive than other cell types to ROS [6]. These observations have prompted researchers to determine whether an enhanced antioxidant capacity could protect β-cells against ROS. In insulin-producing cell lines, enhancing the expression of antioxidant enzymes alleviates ROS-induced toxicity [7, 8]. In diabetic rodent models, antioxidant drugs facilitate the preservation of β-cell mass and insulin content [8–10]. In transgenic animal models, β-cell-specific overexpression of antioxidants protects islets from acute ROS-induced damage [11–16]. In contrast, antioxidant reduction makes β-cells sensitize to ROS [17–19]. However, the metabolic outcomes of different antioxidant transgenic models may vary largely, including opposing roles on insulin sensitivity and β-cell function [16, 20, 21]. Importantly, in clinical trials, antioxidant supplementation is still inconclusive in diabetes prevention [22, 23]. Therefore, the complexity of ROS/RNS and antioxidant defense system in pancreatic β-cells and other tissues in rodents and humans may also be various. Thus, the study using transgenic animal models with antioxidant overexpression in pancreatic β-cells might provide some new evidences to β-cell defense system.

Metallothionein (MT) is a family of low-molecular weight, cysteine-rich, metal-binding proteins. Besides its important role in zinc homeostasis, MT also acts as a potent antioxidant that protects cells and tissues from oxidative stress [24–26]. These functions are related to the presence of abundant cysteine residues. Compared to other antioxidants, MT is a potent antioxidant against a wide range of free radicals, including nitric oxide, peroxynitrite, hydrogen peroxide, superoxide and streptozotocin (STZ). In humans, polymorphisms in the MT-encoding genes *MT1A* and *MT2A* have been shown to be associated with a higher risk to promote the development of T2D and diabetic complications [27,28]. Pancreatic β-cell-specific MT-tg in FVB mice showed no impaired islet insulin secretion [13]. When transferring MT-tg to an NOD background, however, MT-tg greatly accelerated the development of diabetes after cyclophosphamide treatment in male NOD mice [29]. NOD mouse is a model of diabetes but C57BL/6J mouse is not, however, diabetes can be induced by high fat diet in C57BL/6J mouse. We hypothesized that MT-tg overexpression in pancreatic β-cells would prevent ROS damage and preserve β-cell function in C57BL/6J mice. In present study, pancreatic β-cell-specific MT-tg was transferred to C57BL/6J background, and β-cell function, islet morphology, and specific gene expressions in MT-tg mouse model were investigated.

## Materials and Methods

### Ethics statement

The study was conducted in strict compliance with the principles of animal laboratory care under the guidelines of both the National Institutes of Health (NIH) and the Louisiana State University Health Science Center and Research Institute for Children’s Institutional Animal Care and Use Committees (IACUC, New Orleans, LA, USA). The IACUC approved the animal protocols used in the present study (Protocol Number: IACUC 105). All surgical procedures were performed under anesthesia introduced by intraperitoneal injection with ketamine (100 mg/kg body weight) and xylazine (10 mg/kg body weight).

### Generation of transgenic MT mice with C57BL/6J background

Pancreatic β-cell-specific MT-tg in FVB background was provided by Dr. Paul Epstein in the University of Louisville and described [13]. Briefly, the transgene contains a human insulin promoter, as well as all exons and introns of the human *MT2A* gene (NM_005953). The transgenic MT showed no impaired effect on β-cells. The selected transgenic HMT-2 line possessed a 30-fold higher expression of MT. When this line was transferred to an NOD background, both immunohistochemical analysis of MT expression in pancreas sections and western blot analysis of the MT protein in various tissues demonstrated that the expression of MT-tg was islet-specific [29]. In present study, MT-tg in FVB mice was transferred to a C57BL/6J background by backcrossing with C57BL/6J mice (The Jackson Laboratory, Bar Harbor, ME, USA) for at least 7 successive generations. In each generation, offspring were genotyped using tail DNA and detected by quantitative real-time PCR (qPCR) analysis to determine the presence of transgenic alleles. The mice were maintained at 25°C with a 12 h light and 12 h darkness cycle and received *ad libitum* standard chow and water. Body weight was measured at the day of weaning (3-week-old), and at 8, 12, 16, and 18 weeks of age. The parameters reflecting pancreatic beta-cell features and function were measured in both MT-tg mice and wild-type littermates (WT) at 8- and 18-week of age in both males and females.

#### *In vivo* treatment with streptozotocin

Streptozotocin (STZ) (Sigma, Milwaukee, WI, USA) was dissolved in sterile citrate buffer (0.05 M sodium citrate, pH 4.5) and used within 5 min of preparation. Male and female MT-tg and WT mice were treated with a single dosage of STZ (200 mg/kg) by intraperitoneal injection at 8 weeks of age (n ≥ 7 in each group). After STZ administration, non-fasting blood glucose levels were monitored as indicated in Results.

### Glucose tolerance test and insulin assay

Tail snipping was performed to collect blood and blood glucose was measured by using a glucose analyzer (Analox Instruments, Lunenburg, MA, USA). After 5 h fasting, intraperitoneal glucose tolerance test (IPGTT) was performed with 2.0 g glucose per kg body weight in both MT-tg mice and WT littermates at 8 weeks and 18 weeks of age (n ≥ 5 in each group). Blood glucose levels were determined at 0, 15, 30, 60, and 120 min, respectively, and blood samples were collected (at 0, 15, and 30 min only) for measuring plasma insulin levels. Insulin level was measured by using an Ultrasensitive Mouse Insulin-ELISA Test Kit (Mercodia, Uppsala, Sweden).

### Pancreatic islet isolation and morphology assessment

Islets were isolated from mice using an adaptation of the Gotoh method [30]: pancreatic duct infiltration with collagenase, Histopaque^TM^ gradient separation, and hand picking. Before performing measurements, islets were cultured for 1 h at 37°C in humidified air and 5% CO2 in RPMI 1640 supplemented with 5.5 mM glucose, and 10% newborn calf serum, 2mM glutamine, 100 U/ml penicillin, 0.1 mg/ml streptomycin (all from Gibco, Grand Island, NY, USA). For morphology assessment, freshly isolated islets were immersed in RPMI 1640 supplemented with 10% fetal bovine serum, and the pictures were taken within 1 hour under a light microscope (CX21, Olympus America Inc., Melville, NY, USA). The surface area of islets was quantified using an image analysis softeware ImageJ (http://imagej.nih.gov/ij/).

### Pancreatic insulin content measurement

The pancreas (only males) (n ≥ 4 in each group) was isolated, weighed, extracted with acid ethanol, and measured by using an insulin assay (Mercodia, Uppsala, Sweden). Protein concentrations were determined by using the BioRad protein assay (BioRad, Hercules, CA, USA) using bovine serum albumin as a standard and used to normalize insulin content.

### Islet DNA, protein and insulin contents

Twenty isolated islets (duplicate) were sonicated in 0.2 ml of PBS; 50 ul of islet homogenate was used for protein or DNA assays. DNA was measured by the Labarca method [31], and islet protein and insulin contents were measured as described above.

### Insulin secretion

Insulin secretion was performed as described previously [32]. Briefly, 10 islets were cultured in each 5 ml vial with 1ml KRB (Krebs–Ringer bicarbonate buffer supplemented with10 mM HEPES, pH7.4 and 0.1% BSA, bubbled with5% CO2, 95% O2) containing 2.8, 16.7 mM glucose or 16.7 mM glucose plus 10 mM L-arginine in a 37°C shaking water-bath. After incubation for 60 min, vials were moved from the water-bath to ice to stop the reaction, followed by a brief centrifugation at 4°C, 0.5ml KRB was moved into a glass tube and stored at −20°C pending assay for insulin as described above.

### Quantitative real-time PCR and PCR array analyses

Islets were isolated from MT-tg (n = 4) and WT (n = 6) male mice at 8 weeks of age. Total RNA was extracted from each islet using an RNeasy plus Mini kit (QIAGEN Inc., Valencia, CA, USA) and reverse transcribed using Superscript III First-Synthesis System for RT-PCR (Invitrogen, Carlsbad, CA, USA) according to the manufacturer’s recommendations using oligo(dT) primers. Quantitative PCR was performed in duplicate using SYBR Green Master Mix (SABiosciences, Frederick, MD, USA) on iCycle (Bio-Rad Laboratories, Hercules, CA, USA) with gene specific primers for *transporter type 2* (*Glut2*), *glucokinase* (*Gck*), *pancreatic and duodenal homeobox 1* (*Pdx1*), *mitochondrial uncoupling protein 2* (*Ucp2*), and *forkhead box 1* (*Foxo1*). The presence of single PCR products was verified by melt-curve analysis and agarose gel electrophoresis. The amplification efficiency of each gene was calculated by using the LinRegPCR (11.0) program [33] with background-subtracted fluorescence data provided by the iCycle software. The selection of appropriate reference genes was performed using geNorm [34] among five candidates, which included *peptidylprolyl isomerase A* (*Ppia*), *ribosomal protein L13a* (*Rpl13a*), *beta-actin* (*Actb*), *β 2 microglobulin* (*B2m*), and *18S ribosomal RNA* (*Rn18s*) gene. Gene expression was normalized by the geometric means of two selected reference genes, *Actb* and *Rpl13a*. The primer sequences for the 5 studies and 5 reference genes are listed in Table 1.

**Table 1:** Sequences of primers for RT-PCR used in this study.

Gene	Primer sequence (5'→3')		Amplicon (bp)
*Glut2*	Forward	CTGTGTCCAGCTTTGCAGTG	103
	Reverse	GAGGGCTCCAGTCAATGAGA	
*Gck*	Forward	GGCTTCACCTTCTCCTTCCC	128
	Reverse	TGTTGTTCCCTTCTGCTCCG	
*Pdx1*	Forward	GGCTCGCCCATCCACCT	146
	Reverse	GTAAGCACCTCCTGCCCACT	
*Foxo1*	Forward	GTGGATGGTGAAGAGCGTGC	94
	Reverse	AAGGGACAGATTGTGGCGAA	
*Ucp2*	Forward	AAGACCATTGCACGAGAGGA	139
	Reverse	TGAGGTTGGCTTTCAGGAGA	
*B2m*	Forward	CTGGTCTTTCTGGTGCTTGTCT	104
	Reverse	TATGTTCGGCTTCCCATTCTC	
*Actb*	Forward	TACAATGAGCTGCGTGTGGC	125
	Reverse	ATGGCTGGGGTGTTGAAGGT	
*Rn18s*	Forward	AAATAGCCTTCGCCATCACTG	172
	Reverse	GCTCCACCTCATCCTCCGT	
*Rpl13a*	Forward	ATGGTGGTCCCTGCTGCT	143
	Reverse	GCCTTTTCCTTCCGTTTCTC	
*Ppia*	Forward	AGCATACAGGTCCTGGCATC	127
	Reverse	TTCACCTTCCCAAAGACCAC	

For PCR array analysis, islets were derived from the mixture of 3 MT-tg or 3 WT male mice. Approximately 500 ng of total RNA from each group extracted by using the RNeasy plus Mini kit was reverse transcribed to cDNA using the RT^2^ First Strand kit as per manufacturer’s instructions (SABiosciences, Frederick, MD, USA). For each 96-well plate, 25 μL of a mastermix containing cDNA and pathway RT^2^ Profiler PCR plate (SABiosciences) was prepared. The Mouse Diabetes PCR Array (PAMM-023A, SABiosciences) was used. Amplification was performed in accordance with the manufacturer’s guidelines. For data analysis, four housekeeping genes, *beta-glucuronidase* (*Gusb*), *hypoxanthinephosphoribosyltransferase 1* (*Hprt1*), *heat shock protein 90-beta* (*Hsp90ab1*) and *Actb* were used for normalization. The cycle threshold (C_T_) was determined for each sample and normalized to the average C_T_ of the four housekeeping genes. Comparative C_T_ method was used to calculate relative gene expression. Data were represented as fold change relative to that of the control.

### Immunohistochemical analysis

Pancreas sections were prepared from MT-tg (n = 5) and WT male mice (n = 5) at 8 weeks of age. At least three slides from each mouse were examined. Immunostaining was conducted using routine procedures for paraffin embedding and sectioning as described previously [32]. Briefly, sections were deparaffinized, rehydrated, pretreated with target antigen retrieval solution, washed with PBS, blocked with serum, and incubated with primary antibodies [guinea pig anti-insulin (Abcam, Cambridge, MA, USA), diluted to a ratio of 1:50; mouse anti-horse MT (Dako, Carpinteria, CA, USA), diluted to 1:50, or rabbit anti-glucagon (Abcam), diluted to 1:50; goat anti-PDX1, diluted to 1:50 (Abcam)] at 4°C overnight. After incubation, immune complexes were detected by double-labeling immunohistochemistry, incubation with fluorescein isothiocyanate (FITC)-labeled anti-guinea pig IgG, and Cy3-conjugated anti-mouse IgG or Cy5-conjugated anti-rabbit IgG and Cy3-conjugated anti-goat IgG (1:200; all from Jackson ImmunoResearch Laboratories, West Grove, PA, USA).

### Data presentation and statistical analysis

All data were expressed as means ± SEM. The student’s *t*-test was used to compare data between two groups. P < 0.05 was considered statistically significant. P values are provided in the figure legends and are marked by asterisks within the figures. Statistical analysis was performed using SigmaStat software.

## Results

### Pancreatic β-cell-specific overexpressing MT was successfully transferred to a C57BL/6J background

**Fig 1:**
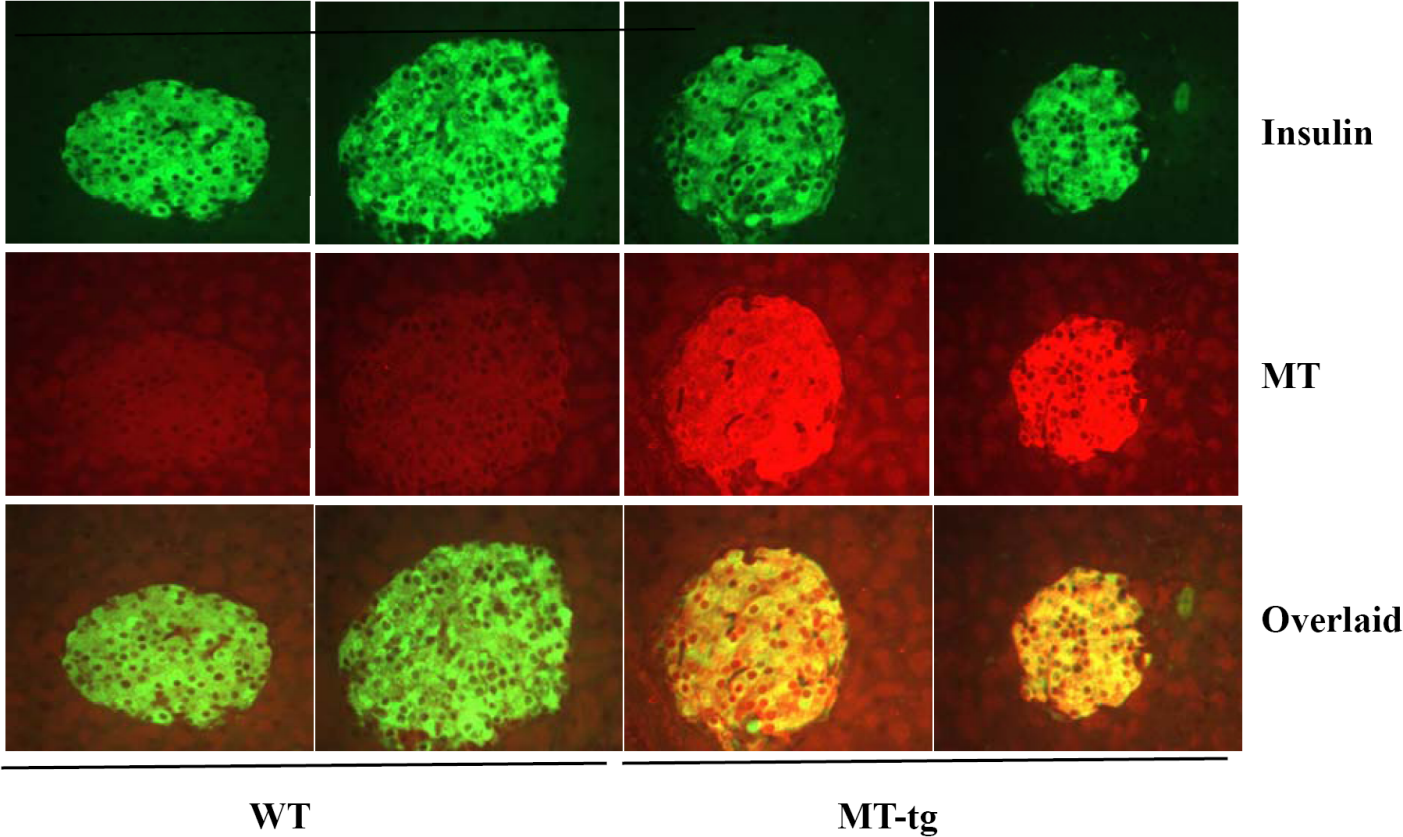
MT overexpression in pancreatic islets of transgenic C57BL/6J mice. MT (red) and insulin (green) immunostaining of islets of MT-tg mice and its control littermates is indicated in different colors. MT staining coincided with insulin staining is indicated by overlaid images.

By backcrossing with C57BL/6J mice for at least 7 successive generations, β-cell-specific overexpressing MT was successfully transferred to a C57BL/6J background. As shown in Fig 1, the transgenic protein was highly expressed in pancreatic islets. No differences were observed in body weight at 3, 8, 12, 16, and 18 weeks of age in both MT-tg and WT mice (data not shown). These observations suggest that MT-tg does not influence development and growth.

### MT overexpression protected adult mice from acute ROS damage *in vivo*

**Fig 2:**
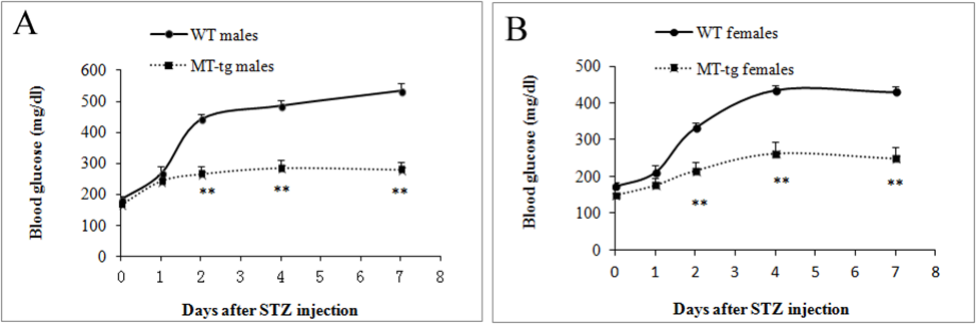
Blood glucose levels in MT-tg and WT mice after *in vivo* STZ treatment. (A) MT-tg (n = 8) and WT (n = 7) male mice; (B) MT-tg (n = 13) and WT (n = 21) female mice. * p<0.05, ** p<0.01 *vs* WT.

To test whether transgenic MT prevents acute ROS damage in mice, *in vivo* STZ treatment was performed. After STZ treatment, blood glucose values, which were monitored for the next 6 days, indicated that the onset of diabetes was significantly delayed in both MT-tg males and females (Fig 2A and 2B).

### MT overexpression impaired β-cells, and the damage was more severe in male mice

**Fig 3:**
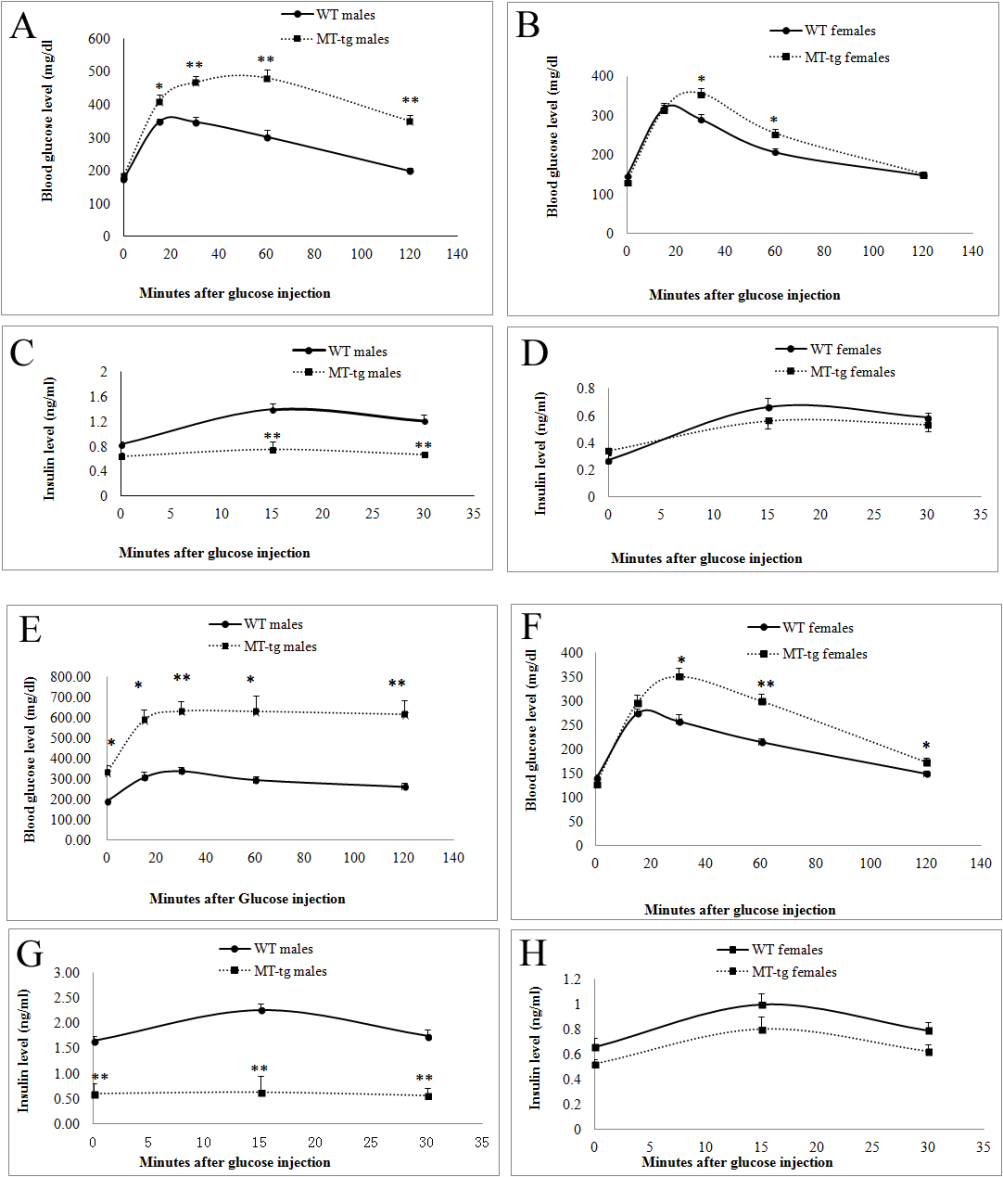
Glucose tolerance tests (GTT) and plasma insulin levels during GTT in 8- and 18-week-old MT-tg and WT mice. **(**A, C) 8-week old MT-tg (n = 10) and WT (n = 14) male mice; (B, D) 8-week old MT-tg (n = 8) and WT (n = 10) female mice; (E, G) 18-week old MT-tg (n = 5) and WT (n = 5) male mice; (F, H) 18-week old MT-tg (n = 8) and WT (n = 10) female mice. * p<0.05, ** p<0.01 *vs* WT.

IPGTT was performed in both MT-tg and WT mice at 8 weeks and 18 weeks of age, respectively. At 8 weeks of age, MT-tg male mice developed severe glucose intolerance (Fig 3A), and MT-tg impaired glucose stimulated insulin secretion (GSIS) (Fig 3C) during GTT. MT-tg female mice also showed glucose intolerance (Fig 3B) and reduced insulin secretion (Fig 3D) during GTT, but much less severe than those observed in males. At 18 weeks of age, all MT-tg male mice developed significant diabetes; fasting blood glucose levels (Fig 3E, zero time) in MT-tg male mice were significantly higher than those observed in WT, and fasting plasma insulin levels (Fig 3G, zero time) in MT-tg male mice were much lower. MT-tg pancreatic β-cells in males demonstrated no response to high glucose during IPGTT (Fig 3G). MT-tg female mice also developed severely impaired glucose tolerance (Fig 3F), but the levels of fasting blood glucose (Fig 3H, zero time) remained similar to those observed in WT littermates. These data show that MT-tg overexpression induces β-cell impairment, and more severe damage occurs in males.

### Pancreatic insulin content decreased in MT-tg mice

To determine whether impaired insulin secretion in MT-tg mice was associated with islet insulin synthesis, whole pancreatic insulin content was measured in 8- and 18-week-old mice. Our data (Table 2) show that pancreatic insulin content was decreased in both male and female MT-tg mice, and this reduction was more significant in males and worsened with age. These findings indicate that islet insulin synthesis was also impaired in MT-tg mice.

**Table 2:** Pancreatic insulin content in MT-tg and WT mice.

Group	Males (ug/gram protein)		Females (ug/gram protein)
	8-week-old	18-week-old	8-week-old
WT	2.80 ± 0.23 (n = 5)	4.10 ± 0.61 (n = 5)	4.78 ±1.05 (n = 5)
MT-tg	1.68 ± 0.21 (n = 4)*	1.33 ± 0.29 (n = 8)**	2.17 ± 0.24 (n = 4)

Note: Values are expressed as the mean ± SEM. n values represent the number of mice. 18-week-old female pancreas was not prepared.

* p < 0.05

** p < 0.001 *vs* WT.

### Insulin secretion in islets of MT-tg mice

**Fig 4:**
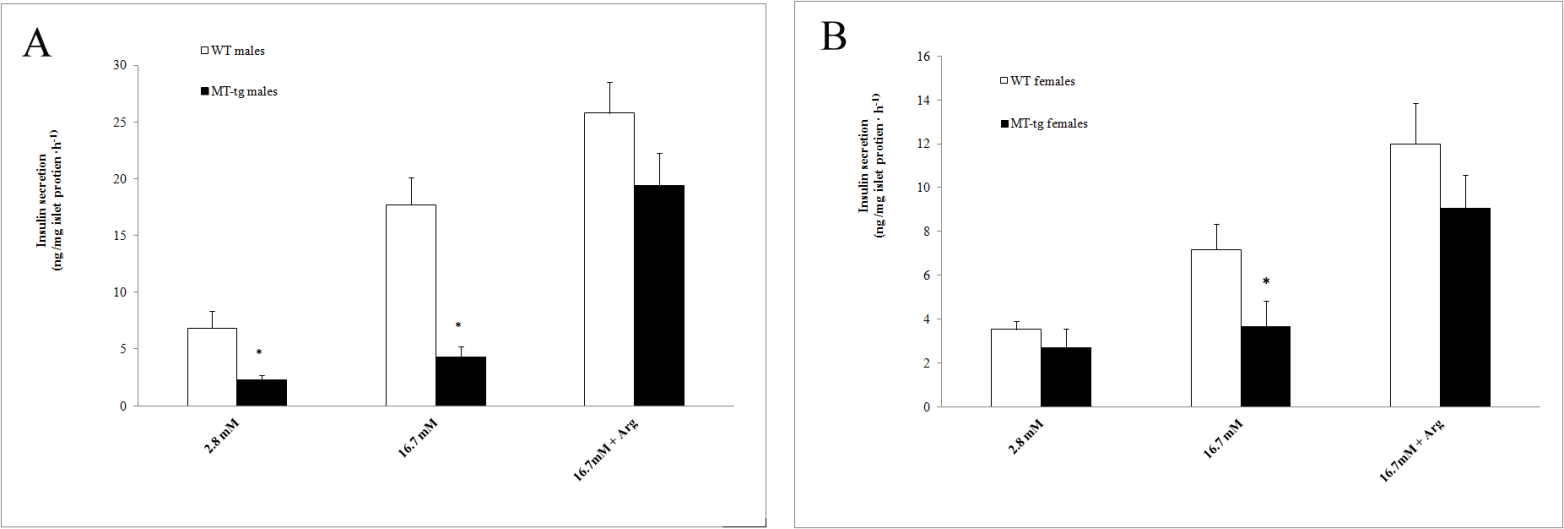
Insulin secretion from islets of 8-week-old MT-tg and WT mice. **(**A) MT-tg (n = 3) and WT (n = 3) male mice; (B) MT-tg (n = 5) and WT (n = 5) female mice. 2.8 mM = 2.8 mmol/l, 16.7 mM = 16.7 mmol/l glucose concentration in KRB, respectively; Arg = 10 mmol/l L-arginine; * p<0.05 *vs* WT.

To determine the impact of MT-tg overexpression on insulin release, insulin secretion was assayed in islets isolated from 8-week-old MT-tg and WT mice. The results showed that MT-tg islets didn’t response to high glucose stimulation (16.7 mM) and had significant reduction in insulin secretion compared with WT mice (Fig 4A and 4B). The reduction was also observed in MT-tg male mice under basal glucose level (Fig 4A, 2.8 mM glucose). These are consistent with both IPGTT test and plasma insulin levels shown in Fig 3, indicating β-cell desensitization in MT-tg mice. Interestingly, in the presence of 16.7 mM glucose plus 10 mM L-arginine, a much more increase of insulin secretion (above basal level) was observed in MT-tg male mice, indicating that β-cell glucose potentiation was only slightly impaired in MT-tg mice at 8-week of age.

### Abnormal morphology and contents in islets isolated from MT-tg male mice

**Fig 5:**
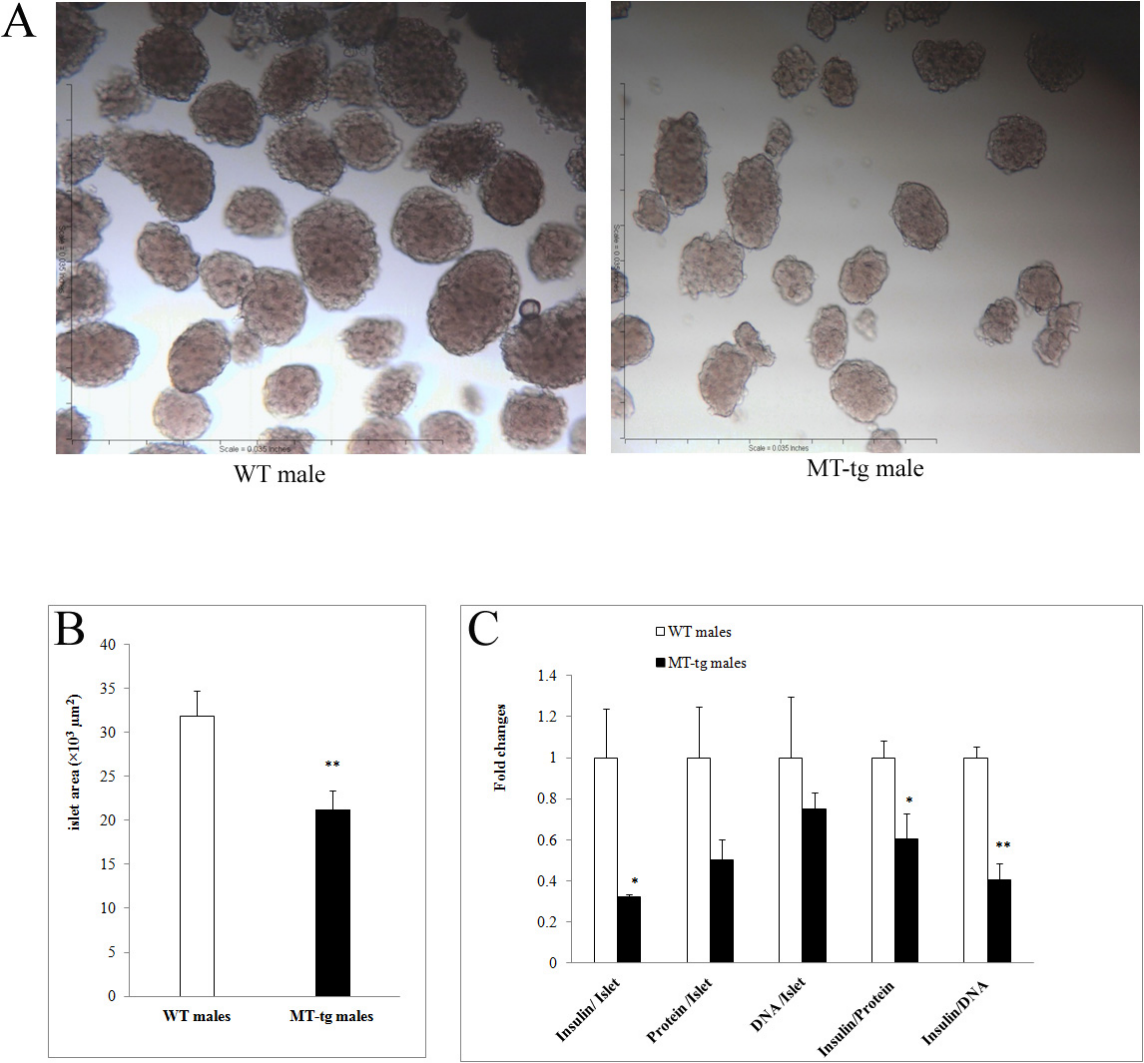
Islet morphology, size, DNA, protein and insulin contents in 8-week-old MT-tg and WT male mice. (A) represents images of islet under a microscope; (B) quantified islet size; (C) islet DNA, protein and insulin contents. * p < 0.05, ** p < 0.001 *vs* WT.

MT-tg male isolated islets obviously appeared different from their WT littermates. Under a light microscope, isolated islets showed that the size of MT-tg islets was smaller (Fig 5A, right panel), and a lower number of islets were isolated from the MT-tg mice (80–120 in MT-tg *vs*. 150–200 in control). The average size of islets (islet area) from MT-tg mice was only 59%-73% of those in WT islets (Fig 5B). Islet protein content and insulin content were significant reduced in MT-tg male mice (Fig 5C). The reductions are consistent with the smaller size and lower density in insulin staining of MT-tg mice (Fig 1), indicating that MT overexpression affects not only β-cells function but also islet morphological features and insulin synthesis.

### Alteration of gene expression in MT-tg mice

**Fig 6:**
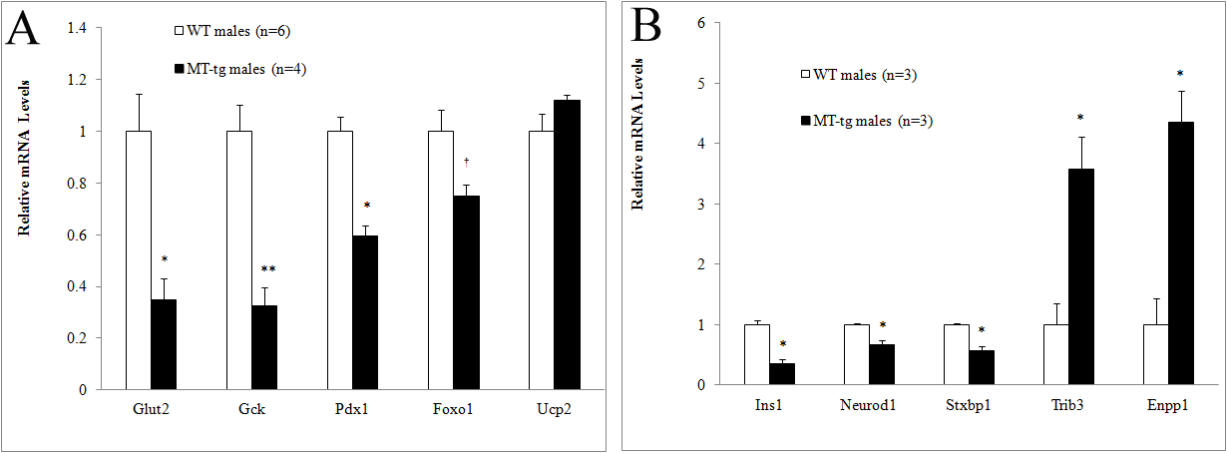
Relative mRNA levels in islets of MT-tg and WT male mice. **(**A) using regular RT-PCR, the value of each gene was normalized to *Actb* and *Rpl13a*. (B) using PCR Array, the value of each gene was normalized to *Gusb*, *Hprt1*, *Hsp90ab1* and *Actb*. The gene expression level in MT-tg islets was presented as fold relative to WT (set as 1). Data are mean ±SEM. n values represent the number of mice. * p <0.05, ** p <0.001, ^†^ p = 0.053 *vs* WT.

To identify the underlying molecular mechanism of reduced islet insulin synthesis and impaired GSIS in MT-tg mice, the gene expression of *Glut2*, *Gck*, *Pdx1*, *Foxo1*, *Ucp2*, and other 84 genes related to the onset, development, and progression of diabetes were assessed in 8-week-old male mice. Downregulation of *Glut2*, *Gck*, and *Pdx1* was observed in MT-tg male mice (Fig 6A). *Foxo1* expression also showed a decreasing trend (p = 0.052). Expression of *Ucp2* in MT-tg male mice was similar to those in WT mice. Among 84 genes evaluated by PCR array, except for 18 genes that expression levels were too low (the average CT values > 35) and 61 genes that expression levels showed no statistical differences, 5 genes showed statistically significant changes in expression levels (Fig 6B) (S1 Table). The level of expression of *insulin 1* (*Ins1*), *neurogenic differentiation factor 1* (*NeuroD1*), and *syntaxin-binding protein 1* (*Stxbp1*) was reduced, whereas that of *tribbles homolog 3* (*Trib3*) and *ectonucleotide pyrophosphatase/phosphodiesterase 1* (*Enpp1*) significantly increased. These observations suggest that alterations in the expression of genes in MT-tg islets might relate to the reduction of insulin synthesis and impaired GSIS in MT-tg mice, and might also involve in glucose transport and sensing, insulin exocytosis, and transcriptional regulations.

### PDX1 expression level decreased in MT-tg mice

**Fig 7:**
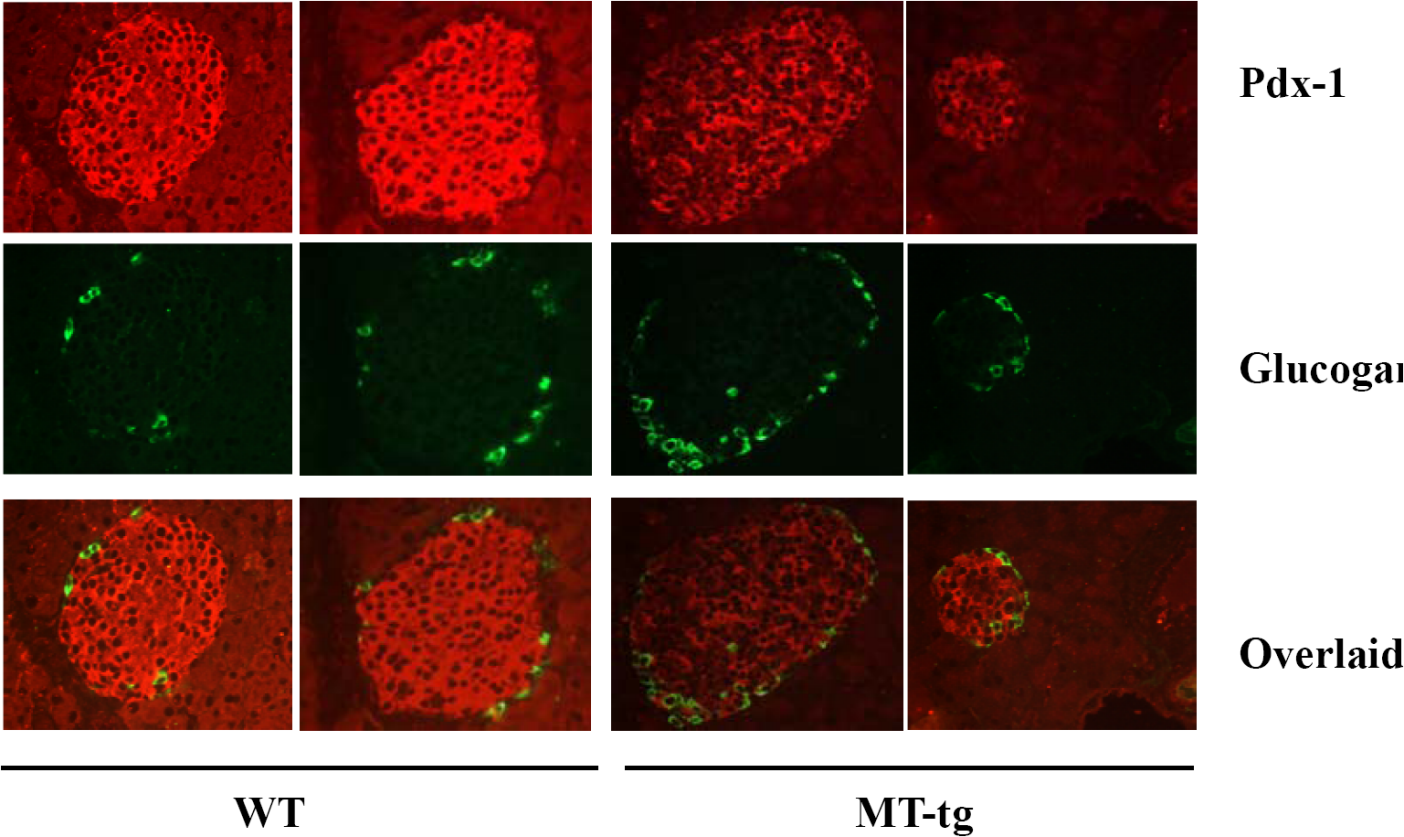
Decreased PDX1 level in pancreatic islets of MT-tg mice. PDX1 (red) and glucogan (green) immunostaining in islets of 8-week-old MT-tg and WT male mice.

PDX1 is a critical regulator of pancreatic β-cell differentiation, survival, and insulin synthesis. RT-PCR indicated that the mRNA level of *Pdx1* was decreased in MT-tg islets. To confirm the RT-PCR results, PDX1 immunostaining was performed, which showed that the PDX1 expression level was lower in MT-tg mice (Fig 7).

## Discussion

In the present study, C57BL/6J mice that carry MT-tg specifically overexpressed in pancreatic β-cells were generated. We hypothesized that MT-tg in pancreatic β-cells could prevent damage from ROS and preserve β-cell function in this model. The study showed that the MT-tg delayed the onset of diabetes in STZ *in vivo* treated adult mice; unfortunately, however, MT overexpression accelerated the development of diabetes in standard chow diet fed adult mice, pancreatic β-cell function was also significantly impaired in these mice, and more severe diabetes was demonstrated in males. The underlying mechanism by which MT-tg impairs pancreatic β-cell function and induces diabetes is not clear; however, the present study and other related reports may provide some clues.

### Genetic background might play an important role in the diverse effect of antioxidants on pancreatic β-cells

The pancreatic β-cell-specific MT-tg was originally generated in FVB mice that were not showed to impair islet insulin secretion [13]. When transferred to an NOD background, an animal model that is susceptible to spontaneous development of T1D, MT overexpression greatly accelerated the onset of diabetes after cyclophosphamide treatment and also accelerated spontaneous diabetes in male NOD mice [29]. In the present study, when β-cell MT-tg was transferred to C57BL/6J mice, an animal model that is susceptible to high fat diet-induced obesity and T2D, significant β-cell impairment was observed in standard chow diet fed adults, and more severe β-cell damage was showed in males. Different responses to the same transgenic antioxidant were also observed in β-cell-specific catalse overexpressed mice, which had the same detrimental effects in NOD mice as did MT overexpression [29]; in FVB mice, it showed no impaired islet insulin secretion [13]. Different responses were also observed in Gpx1 overexpression models [16]. Total islet volume and β-cell volume did not differ between transgenic mice and controls on the C57BLKS/J background; however, when backcrossed with db/db mice, overexpressing Gpx1 prevented the loss of β-cell volume and insulin granulation. Mice carrying the same genetic modifier exhibiting different phenotypes were also observed among C57BL/6, 129Sv, and DBA backgrounds [35]. This phenotypic heterogeneity indicates that genetic background might play an important role in the effect of antioxidants on β-cell functions. The different genetic components between sexes might also contribute to more severe β-cell impairment in transgenic males that have insufficient amounts of estrogen to protect pancreatic β-cells [36].

### The strong scavenging capacity of MT overexpression prevents islets from acute STZ-induced damages

MT is a group of intracellular metal-binding and cysteine-enriched proteins that is highly inducible in various tissues in response to different types of stress. Four isoforms of MT have been characterized, and MT-1 and MT-2 are the major isoforms in various human and animal tissues, including the pancreas. Although it mainly acts as a regulator of metal homeostasis such as zinc and copper in tissues, MT also acts as a potent antioxidant that protects cells and tissues from oxidative stress. MT is distinct from other antioxidants due to its wide range of scavenging targets, including ROS (O2^-^, H2O2, and HO^-^) and RNS (NO and ONOO^-^) and its high scavenging efficiency [13, 24–26]. The selected transgenic MT line in the present study is characterized by a 30-fold increase in MT expression compared to controls. The high MT expression level, together with the wide range of scavenging targets and the high scavenging efficiency of MT, renders transgenic islets with highly effective scavenging capacity. The finding that MT overexpression effectively prevents acute STZ-induced ROS damage *in vivo* in 8 weeks old mice in this study suggests that MT-tg has strong scavenging capacity.

### MT overexpression might interfere with ROS signals

Although excessive ROS production can directly or indirectly damage cellular macromolecules, a low level of ROS has been suggested to mediate a diverse range of physiological responses, including GSIS [3, 6], hypothalamic glucose sensing [37], and insulin signaling [5]. For these possible pivotal roles of ROS, MT overexpression might interfere with ROS signals, resulting in impaired insulin secretion and insulin resistance during growth and aging. For instance, H_2_O_2_, which is derived from glucose metabolism, has been suggested to be a mediator of GSIS. ROS may also be involved in Ca^2+^ influx, a major event to activate the insulin exocytotic machinery in the early phase of GSIS [3, 4, 38]. The strong scavenging capacity of MT overexpression in this study might interfere with ROS signaling in GSIS and finally result in impaired GSIS. However, it’s of great interest that L-arginine-mediated glucose potentiating in MT-tg male mice was preserved, although GSIS was slightly impaired. Indeed, L-Arginine has been recognized as one of the most powerful insulin secretagogues, although the underlying mechanism has not been fully elucidated. Considerable interests have focused on the possible significance of L-arginine-derived nitric oxide (NO) [39]. It has been shown to be involved, or may not to be involved, or to be a negative modulator in L-arginine mediated GSIS. Because NO is one of the scavenged target of MT, the greater impact of L-arginine on GSIS in MT-tg mice in this study is unlikely contribute to L-arginine-derived NO. The evidences suggest that L-arginine may stimulate membrane depolarization and stimulate Ca^2+^ influx [40], this may be one of the mechanisms by which L-arginine potentiates GSIS in MT-tg male mice.

### Unique gene expression pattern in MT-tg mice suggests that MT overexpression has a unique diabetogenic mechanism in C57BL/6J mice

Gene expression analysis has demonstrated alterations in several important genes involving glucose metabolism and insulin regulation in MT-tg mice. For the crucial role of PDX1 in controlling pancreatic development, β-cell differentiation, survival, and insulin synthesis [41, 42], severely reduced *Pdx1* gene expression and PDX1 protein level may explain a significant β-cell dysfunction and abnormal islet morphology in MT-tg mice. The expression level of NeuroD, another important transcription factor in pancreatic β-cell differentiation and mature [42, 43], was also reduced. On the other hand, the expression of *TRIB3* was significantly enhanced, which suggests that *TRIB3* overexpression is associated with impaired insulin exocytosis [44] and increased β-cell apoptosis [45]. *Stxbp1*, a gene that plays essential roles in insulin exocytosis [46], was downregulated in transgenic mice. The expression levels of two genes (*Stxbp1* and *TRIB3*) involved in insulin exocytosis were reduced, indicating that insulin exocytosis in MT-tg mice might be impaired. The role of *Enpp1* in β-cells remains elusive, although its overexpression in plasma has been associated with insulin resistance and T2D [47], and the *in vitro* studies indicate that it affects both β-cell function and survival [48]. Because PDX1 regulates various genes, including *Ins1*, *Gck*, and *Glut2*, its downregulation would decrease the expression levels of Ins1, Gck, and Glut2. The observed reduction in PDX1 expression level was consistent with the findings in NOD mice that showed a decrease in the activation of the Akt/Pdx1 pathway [29]. Interestingly, *Ucp2*, which encodes a mitochondrial protein that plays a key regulatory role in ROS signaling and GSIS [49], was not affected in the present study. In contrast, it has been reported that increased *Pdx-1* and reduced *Ucp2* expression were observed in β-cell-specific Gpx1-overexpressing mice [20, 21]. The unique alteration of gene expression pattern in the present study suggests MT-tg overexpression might be involved in a unique diabetogenic mechanism in C57BL/6J mice; therefore, further studies will be performed to explore this mechanism.

In conclusion, pancreatic β-cell specific MT overexpression in C57BL/6J mice protected β-cells from acute ROS-induced damage at young age, whereas it impaired GSIS and promoted the development of diabetes in adult C57BL/6J mice, and more severe damage was found in males. The present study indicates that a long-term application of antioxidant MT offers no protection to pancreatic β-cells, and it eventually impairs β-cell functions.

## Supporting Information

S1 Table Relative mRNA levels of 84 genes in islets of MT-tg and WT male mice by using PCR Array. The value of each gene was normalized to *Gusb*, *Hprt1*, *Hsp90ab1* and *Actb*. The gene expression level in MT-tg islets was presented as fold relative to WT (set as 1). N/A represents the average CT values > 35.
(XLSX)
